# Stereotactic body radiotherapy for intramedullary metastases: a retrospective series at the Oscar Lambret center and a systematic review

**DOI:** 10.1186/s12885-021-08901-6

**Published:** 2021-10-30

**Authors:** Marion Tonneau, Raphaëlle Mouttet-Audouard, Florence Le Tinier, Xavier Mirabel, David Pasquier

**Affiliations:** 1 Département Universitaire de Radiothérapie – Centre Oscar Lambret, Lille, France; 2 Centre de Recherche du Centre Hospitalier Universitaire de Montréal (CRCHUM), Qc, Montréal, Canada; 3 CRIStAL UMR CNRS 9189, Lille University, Lille, France

**Keywords:** Radiotherapy, Stereotactic, Radiosurgery, SBRT, Intramedullary metastasis, Neuro-oncology

## Abstract

**Background:**

Intramedullary metastasis (IMM) is a rare disease with poor prognosis. The incidence of IMMs has increased, which has been linked to improved systemic treatment in many cancers. Surgery and/or radiotherapy are the most commonly used treatments; only small-sample retrospective studies and case reports on stereotactic body radiotherapy (SBRT) have reported acceptable results in terms of local control and clinical improvement, with no reported toxicity. Thus, we performed this monocentric retrospective study on five cases treated with SBRT for IMMs, which we supplemented with a systematic review of the literature.

**Methods:**

We included all patients treated for IMM with SBRT. The target tumor volume, progression-free survival, prescription patterns in SBRT, survival without neurological deficit, neurological functional improvement after treatment, and overall survival were determined. Results: Five patients treated with a median dose of 30 Gy in a median number of fractions of 5 (prescribed at a median isodose of 86%) included. The median follow-up duration was 23 months. Two patients showed clinical improvement. Three patients remained stable. Radiologically, 25% of patients had complete response and 50% had stable disease. No significant treatment-related toxicity was observed. Conclusion: SBRT appears to be a safe, effective, and rapid treatment option for palliative patients.

## Introduction

The development of intramedullary metastases (IMMs) is a rare event, and IMMs account for 4.2–8.5% of central nervous system metastases [1]. However, improved survival owing to more effective treatments for many cancers is reflected in the increased incidence of IMM. More than half of all cases of IMMs are secondary to lung cancer (54%), breast cancer (11%), renal carcinoma (9%), melanoma (8%), or lymphoma (4%) [1]. These lesions can appear at any time in the history of the oncological disease and can affect the medulla. Because of the low number of lesions treated, therapeutic standards are not clearly defined. Neurosurgery and microsurgical techniques are one of the few available treatments because of their advancements. The application of stereotactic body radiation therapy (SBRT) in the management of spinal lesions has recently emerged, and it was initially used to treat vertebral metastases [2]. It is now considered a safe and effective option [3, 4]. Issues related to myelopathy and radiation-induced spinal cord (SC) injury have historically limited the application of SBRT in treating intramedullary lesions [5, 6]. Thus, there are very few studies on the application of radiosurgery for IMM, and management guidelines have not yet been standardized [7]. In this study, we performed a monocentric retrospective evaluation of the SBRT management of IMM in five patients who were treated with SBRT for IMMs to assess local control and progression-free survival (PFS).

## Materials and methods

### Systematic review

**Fig. 1:**
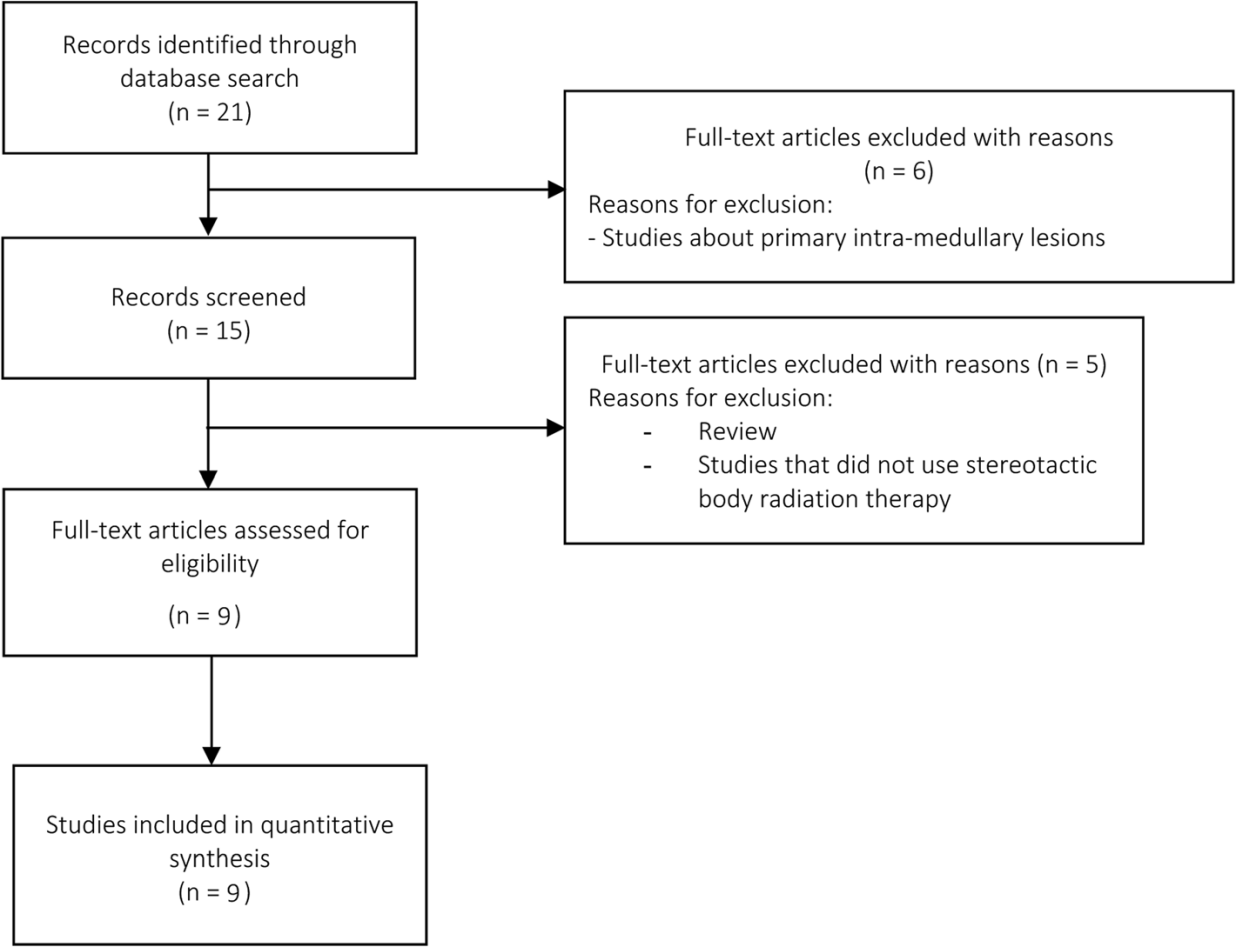
Flowchart showing the process for search and selection of articles

First, we conducted a systematic review of the literature based on the Preferred Reporting Items for Systematic Review and Meta-Analyses (PRISMA) selection method. A systematic review of the scientific literature was conducted using the PubMed, MEDLINE, and Google Scholar databases. The following keywords were used to perform the search: “Radiosurgery,” OR “Stereotactic Radiotherapy,” OR “SBRT,” OR “SABR,” OR “Stereotactic Body Radiotherapy,” OR “Stereotactic Body Radiation Therapy,” AND, “Intra-Medullary Metastasis,” OR “Intra-Medullary Spinal Metastasis,” OR “Intra-Medullary Spinal Cord Metastasis.” No date limit was set. The inclusion criteria were (1) prospective or retrospective studies or case reports, (2) studies including patients with IMM, (3) studies that used SBRT, (4) local control (LC) and PFS reported as the primary or secondary endpoint, and (5) articles published in English or French. The exclusion criteria were (1) use of non-SBRT or hypofractionated radiotherapy, (2) primary intramedullary lesions, and (3) series evaluating only re-irradiation. Information of the following variables was extracted from the studies: type of study, number of patients, total dose delivered, number of fractions, LC at 5 years, clinical and radiological results, and possible toxicities. A flowchart showing the process for the search and selection of the articles is detailed in Fig. 1.

### Population

We retrospectively analyzed patients treated with SBRT for IMM at the Oscar Lambret Center (Lille, France). Five patients were treated between October 2014 and March 2020. The eligibility criteria for their inclusion were as follows: age > 18 years, histologically proven primary cancer, a World Health Organization performance status of ≤2, received SBRT as an ablative therapy using a CyberKnife® linear robotic accelerator, and no previous surgery or radiation therapy for the SC. The cases were discussed by a multidisciplinary team, in consultation with the neurosurgeon, radiation oncologist, and medical oncologist. All patients were treated for IMMs, with the primary goal of symptom palliation or to maximize LC. All patients were treated with SBRT.

### SBRT planning

All treatments were delivered using a high-precision CyberKnife® linear accelerator with 6-MV photons. Treatment simulation was performed using a computed tomography (CT) simulator with millimeter-thickness. The target tumor volume and critical organs, such as the SC, were then determined. Dosimetric parameters were reported according to the ICRU 91 recommendations [8].

### Follow-up and evaluation of response to SBRT

The observation time started in the first fraction of the SBRT. The primary endpoint was PFS, defined as the absence of progression at the treatment site according to the RECIST criteria. The secondary endpoints were the description of survival without neurological deficit, description of neurological functional improvement after treatment, and overall survival (OS). The first clinical follow-up was done at one month. The first radiologic reassessment occurred at three months then every three months. The follow-up was left to the physician’s choice, by CT scan and/or Magnetic Resonance Imaging (MRI), depending on the clinical context.

### Ethics approval

Clear and fair information was provided to all patients according to the recommendations of the Reference Methodology MR-004, which was related to the processing of personal data implemented in the context of research not involving humans, studies, and evaluations in the field of health, on May 3, 2018.

### Statistical analysis

Clinical and dosimetric characteristics were described according to the classical rules of descriptive statistics. In view of the small number of patients, the statistical analysis was essentially descriptive, and no tests were performed. Dosimetry parameters were described in terms of median, extremes, mean, and standard deviation for quantitative data and in terms of frequency and percentage for qualitative data.

## Results

### Systematic review

We identified 9 articles reporting on the treatment of intramedullary metastasis by means of SBRT. Of these, six were case reports [9–14], and three were larger case series [15–17]. Fifty-four patients with a total of 69 intramedullary metastases were included in the analysis. The most common histologies were breast carcinoma (*n* = 21), non-small cell lung cancer (*n* = 8), and clear cell renal carcinoma (*n* = 3) (see Table 1). Of the 69 IMMs, 31 (45%) were in the cervical spinal cord, 25 (36%) spanned over the thoracic cord, 12 (17%) were lumbar et one (1,5%) was located at the conus medullaris. Only four papers [13, 15–17] reported the tumor volume, with a mean value of 0.99 cm^3^. Most of the lesions were treated by means of CyberKnife® (90%), followed by seven (10%) lesions having undergone treatment with LINAC. Radiation total doses ranged from 14 to 39 Gy. Regarding fractionation, they range between 1 to 13 fractions. Treatment outcomes were favorable in most of the studies, with improvement [9, 12, 13, 15–17] or stable symptoms [10, 11, 14–17]. Tumor stabilization or decrease in size was observed in most of the studies [11, 12, 15–17]. In all the papers, clinical and radiological stabilization or improvement was described. Reported OS ranged from 2 to 15 months. No treatment-related complications have been reported. All these data are available in Table 2.

**Table 1:** Systematic review of IMM studies wherein patients were treated with SBRT

Author and year	Study design	Number of patients	Number of MIM treated	Histology	MIMs location
**Shin, 2009 [15]**	Retrospective	6	6	Melanoma (*n* = 1), Breast carcinoma (*n* = 1), Breast invasive ductal cell carcinoma (n = 1), renal cell carcinoma (n = 1), non-small cell lung cancer (n = 1), glioma (n = 1)	Cervical (*n* = 5), Thoracic (n = 1)
**Parikh, 2009 [9]**	Case report	1	1	Renal cell carcinoma	Cervical
**Dewas, 2011 [10]**	Case report	1	1	Pleural mesothelioma	Thoracic
**Lieberson, 2012 [11]**	Case report	1	1	Prostate carcinoma	Conus Medullaris
**Veeravagu, 2012 [16]**	Retrospective	9	11	Breast carcinoma (n = 5), non-small cell lung cancer (n = 2), teratoma (n = 1), Breast infiltrating ductal epithelioid (n = 1)	Cervical (*n* = 7), Thoracic (*n* = 3), Lumbar (n = 1)
**Mori et al., 2016 [12]**	Case report	1	1	Papillary thyroid carcinoma	Cervical
**Garcia, 2016 [13]**	Case report	1	1	Breast ductal carcinoma	Cervical
**Barrie, 2019 [14]**	Case report	1	1	Renal Cell Carcinoma	Cervical
**Ehret, 2021 [17]**	Retrospective	33	46	Breast carcinoma (*n* = 16), lung cancer (*n* = 4), malignant melanoma (n = 3), others (*n* = 10)	Cervical (*n* = 15), Thoracic (*n* = 20), Lumbar (*n* = 11)

**Table 2:** General characteristics IMM studies wherein patients were treated with SBRT

Author and year	Mean tumor volume in cubic centimeter	Treatment modality	Dose/fraction	Median follow-up	Overall survival	Clinical outcome	Radiological outcome	Toxicities
**Shin, 2009 [15]**	1.52	LINAC	14 Gy (10–16 Gy) / 1 fraction	10 months	8 (2–19) months	Improvement: 80%, Stable: 10%, Worse: 10%	Complete: 22%, Partial: 33%, Stable: 33%, Progression: 11%	None
**Parikh, 2009 [9]**	NA	CK	15 Gy/3 fractions	26 months	9,8 months	Improvement	Stable	None
**Dewas, 2011 [10]**	NA	CK	20 Gy / 4 fractions	11 months	NR	Stable	Stable	None
**Lieberson, 2012 [11]**	NA	CK	27 Gy / 3 fractions	3 months	15 months	Stable	Complete	None
**Veeravagu, 2012 [16]**	1.17	CK	21 Gy (14–27 Gy) / 3 fractions (1–5)	NR	2 months	Improvement: 11%, Stable: 44%, NA: 55%	Partial: 22%, Stable: 22%, NA: 78%	None
**Mori et al., 2016 [12]**	NA	VMAT	39 Gy / 13 fractions	5 months	NR	Improvement	Partial	None
**Garcia, 2016 [13]**	0.167	CK	17 Gy / 1 fraction	37 months	8 months	Improvement	Stable	None
**Barrie, 2019 [14]**	NA	CK	25 Gy / 5 fractions	26 months	8 (0–65) months	Stable	Progression	None
**Ehret, 2021 [17]**	1.1	CK	16 Gy (6–24 Gy) / 1 fraction (1–3)	8.5 months	11,7 months	Improvement: 27%, Stable: 30%, Worse: 21%	Complete: 79% patients with follow-up imaging	None
**Our series, 2021**		CK	30 Gy (25–36 Gy) / 6 fractions (5–6)	23 months	NR	Improvement: 40%, Stable: 60%	Complete: 25%, Partial: 50%, Stable: 25%	None

Abbreviations: CK, CyberKnife®; Gy, gray; NA, not available; NR, not reach

### Our study

#### Patients’ characteristics

Patient characteristics are summarized in Table 3. All patients who met the inclusion criteria were included in the study, leading to a total of five patients. The median age was 67 years (range, 33–72 years). Primary cancers were lung adenocarcinoma, malignant melanoma, breast cancer and renal cell carcinoma. Two patients with lung adenocarcinoma were both EGFR negative, ALK negative and KRAS negative. For one of them, PD-L1 expression was 70%. All patients except one had brain and extracerebral metastasis at the time of treatment of the IMM. Two patients had concomitant systemic therapy. One patient had NIVOLUMB for a metastatic melanoma. The other one had TRASTUZUMAB PERTUZUMAB for a metastatic breast cancer. The other one didn’t have concomitant systemic treatment (see Table 3).

**Table 3:** Patients’ characteristics

No. of patients	Age (years)	Sex	WHO*	Primary cancer	Mutations and Biomarkers	Concomitant systemic medication	Level	Presenting symptoms	Muscular strength at diagnosis	Use of corticosteroids	Time between diagnostic to start of SBRT (days)
1	67	M	2–3	Lung, adenocarcinoma	EGFR- / ALK- PD-L1 70%	No	T10	Total paraplegia	0/5	No	14
2	69	F	1	Lung, adenocarcinoma	EGFR- / ALK- / KRAS-	No	T11	Posterior cord syndrome	4/5	No	21
3	72	M	2	Kidney, clear cell carcinoma	x	No	L2	Partial paraplegia	3/5	Yes (1 mg/kg)	6
4	33	M	0	Skin, melanoma	x	NIVOLUMAB	T8	None	5/5	No	33
5	62	F	1	Breast, adenocarcinoma	HER +++ / HR +	TRASTUZUMAB PERTUZUMAB	C4	None	5/5	Yes (1 mg/kg)	44

*World Health Organization (WHO) at the diagnosis of metastasis

Abbreviation: HR: Hormonal Receptors

One cervical lesion, one lumbar lesion, and three thoracic lesions were treated. The median interval from the diagnosis of the primary cancer to the diagnosis of IMM was 66.2 months (range: 19.2–178.8) months.

Two patients initially had paraplegia (complete for one patient), one patient had posterior cord syndrome, and the other two patients did not present with symptoms. Corticosteroid use was reported only for two patients (patients 3 and 5) who received 1 mg/kg for 2 weeks and a progressive decrease. The median delay between diagnostic of MIM to start of the radiotherapy was 21 days (6–44 days). The two patients with the longer delay didn’t have any neurologic symptoms (33 and 44 days) (see Table 3). The average tumor volume was 1.4 cc (standard deviation 0.91 cc), and the largest volume was 2.74 cc (see Table 4).

**Table 4:** Treatment characteristics

No. of patients	Total dose (Gy)	Number of fractions	Fraction (Gy)	BED (α/ß = 10) (Gy)	BED (α/ß = 2) (Gy)	GTV (cc)	PTV (cc)
**1**	30	6	5	45	105.0	1.72	2.62
**2**	36	6	6	57.60	144.0	1.16	1.95
**3**	25	5	5	37.50	87.5	2.74	5.27
**4**	36	6	6	57.60	144.0	0.31	0.62
**5**	30	6	5	45	105.0	1.0	1.58

Abbreviations: Gy, gray; cc, cubic centimeter; BED, biologically effective dose

#### Dosimetry planning

All patients underwent planning CT without intravenous contrast and planning MRI. This MRI was used to aid in the delineation of the macroscopic tumor lesion. The GTV was contoured on the CT-scan with a fusion on contrast-enhanced MRI T1 sequence. No additional margin was added for microscopic spread of disease. A Planning Target Volume (PTV) margin of 1 mm was added to the GTV. A thermoformed mask was used for immobilization. No PRV was used for OAR. Dose was prescribed to 73 to 85%, except for patient one whose prescription was more homogeneous (isodose 97%) due to the infiltrating nature of the tumor which encompassed the entire circumference of the spinal cord. All patients were treated in the supine position. The median total dose delivered was 30 Gy. Among the five patients, two were irradiated using a dose of 36 Gy delivered in six fractions, two others, a dose of 30 Gy in six fractions, and the last one, a dose of 25 Gy in five fractions. The fractionation scheme was left to the discretion of the physician. These are hypofractionation schemes commonly used in other clinical situations. All patients were treated on alternate days. The median biologically effective dose (BED) was 45 Gy, assuming α/ß = 10, and ranging from 37.5 Gy for 5 × 5 Gy to 57.40 Gy for the 6 Gy × 6 fraction schedule. The SBRT treatment schedule and dosimetry characteristics are presented in Table 5.

**Table 5:** Dosimetry parameters

No. Of patients	Near_Max (Gy)	Near_Min (Gy)	D50% (Gy)	D98%_GTV (Gy)	D95%_PTV (Gy)	D99%_PTV (Gy)	Prescription isodose (%)	D2%_Spinal_Cord (Gy)	D98%_Spinal_Cord (Gy)	D50%_SPinal_Cord (Gy)
**1**	30.7	28.8	30.0	29.5	29.1	28.5	97	30.6	0.2	13.6
**2**	44.5	20.8	38.8	26.4	22.8	20.0	80	13.9	0.7	0.8
**3**	29.1	25.1	27.1	26.6	25.3	25.0	85	19.7	0.1	0.2
**4**	42.1	27.8	35.8	32.4	28.9	26.7	85	2.8	0.3	0.4
**5**	41.5	24.1	33.5	28.6	24.7	23.6	73	24.4	0.02	0.4

Abbreviations: Gy, gray; Dx%, dose received in x% of the volume; Near_Max: dose received in 2% of the PTV; Near_Min: dose received in 98% of the PTV

#### Clinical outcome

**Fig. 2:**
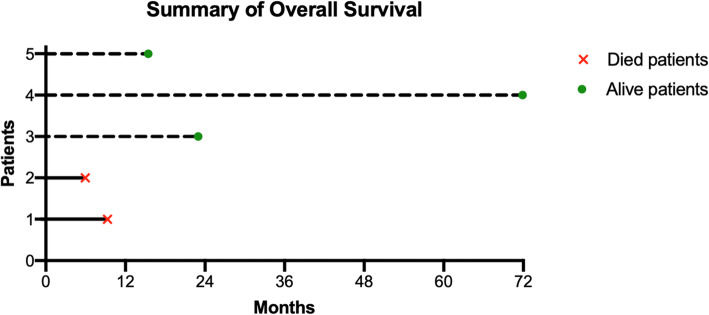
Summary Overall Survival for each patient, in our cohort

The median follow-up period was 23 months (range: 15.4–72 months). OS was heterogeneous between patients, ranged for 5,9 to 72 months. The OS is presented in Fig. 2. The median OS was not reach. Clinical and radiological outcomes are presented in Table 6.

**Table 6:** Clinical and radiological outcomes at first evaluation

No. Of patients	Time to first evaluation	Type of imaging	Motor deficit	Sensory deficit	Clinical response	Imaging	Radiological response
**1**	3 months	Scanner	0	Posterior cord syndrome L1 L–T12 R	Stability	Scan	Complete response
**2**	NA	NA	4	None	Improvement	None	NA
**3**	3 months	Scanner	5	None	Improvement	Scan	Stability
**4**	18 months	MRI	5	None	Stability	MRI	Partial response
**5**	3 months	MRI	5	None	Stability	MRI	Almost complete response

Abbreviations: L: Left, R: Right, MRI: Magnetic Resonance Imaging, NA: Not Available

##### Follow-up

At the end of our study, three patients were still alive, and two patients died of disease progression. No deaths related to the treatment were reported. None of the patients relapsed locally. Two patients presented with distant progression.

##### Radiological response

**Fig. 3:**
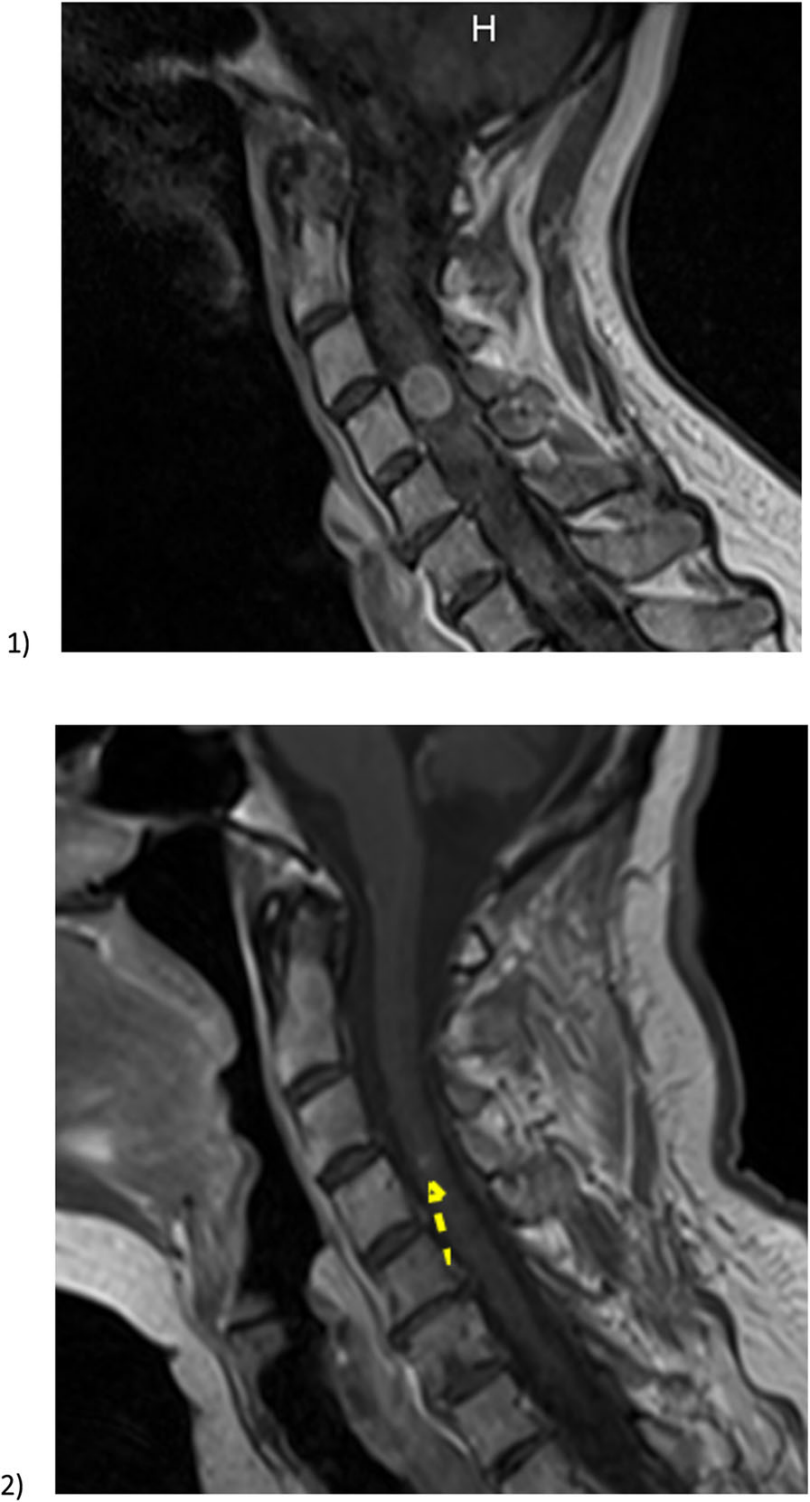
Magnetic resonance imaging (MRI) of patient 5 who was treated with a dose of 30 Gy in five fractions. 1) Pre-stereotactic body radiotherapy (SBRT) MRI with metastasis opposite C4. 2) MRI at 3 months after completing radiotherapy with almost complete response observed

Radiological response to SBRT was evaluated in three patients at 3 months. Two were reassessed with CT and one was reassessed with MRI, according to RECIST criteria. Among these three patients, one had complete response (total dose 30 Gy in 6 × 5 Gy), one had near-complete response (30 Gy in 6 × 5 Gy) (see Fig. 3), and one had lesion stability (25 Gy in 5 × 5 Gy).

For one patient, no dedicated radiological follow-up of the intramedullary lesion was obtainable. This patient was evaluated for the first time, 18 months after treatment, using MRI. This patient had partial response (36 Gy in 6 × 6 Gy) at 18 months.

The last patient died within 3 months of treatment and wasn’t evaluated. The patient who had near-complete response at 3 months, had a complete response on MRI, according to RECIST criteria, at one year.

##### Clinical response

At the first clinical evaluation three patients were stable, and two patients showed functional improvement. The patient with a complete motor deficit did not show clinical recovery during the first reassessment. Only one patient, with partial paraplegia, had complete recovery of motor function. This patient had been treated with 25 Gy in fractions of 5 Gy. The patient who presented with complete recovery of his sensory function had been treated with the 36 Gy in 6 fractions schedule.

#### Toxicities

None of the patients experienced worsening of neurological symptoms during the treatment. The most common side effect was asthenia grade 1/2 (*n* = 2). This occurred during treatment and in the following days. None of the patients developed radiation necrosis, bleeding, myelopathy, acute, or delayed treatment-related complications > grade 1.

## Discussion

In this study, we reported the outcomes of a series of patients treated with SBRT for IMM. Despite the small number of patients, a variety of primary histologies, and different treatment patterns, we reported partial or complete radiological responses in all radiologically reevaluated patients and two clinical improvements (motor deficit), including complete recovery of neurological function with no toxicity.

Three objectives must be considered in the management of these patients: 1) to achieve LC of the tumor to prevent or treat neurological symptomatology, 2) to avoid morbidity in patients who are frequently in palliative care, with limited survival, and 3) to propose a treatment plan adapted to the patient’s general condition. Thus, early diagnosis is important to increasing patient survival and minimizing morbidity. Consequently, the justification of radiosurgery seems attractive in this clinical context. Currently, surgery can be proposed for patients not presenting with distant metastasis, and a histopathological diagnosis is needed [1, 18]. LC after surgical resection is acceptable, but surgery may be responsible for the worsening of neurological symptoms [19]. Currently, there is no standardized SBRT-based treatment for the treatment of IMMs. Several studies have been published on the application of surgery, radiotherapy, or radiosurgery, such as case reports or retrospective studies with a limited number of patients [9–17] (see Table 1). These studies have reported very heterogeneous fractionations adapted to the patient’s irradiation history, tumor radiosensitivity, or local practices (Table 2).

Typically, oligometastatic patients in whom the primary pathology is controlled [19, 20], and with significant or complete neurological deficits are managed surgically [1, 21]. In our review, the tumor volume of treated IMMs averaged 0.9 cc [13, 15–17]. The median tumor volume in our study was 1.4 cc (range: 0.31–2.74 cc), compared to 0.7 cc (range: 0.1–5.8 cc) in the study by Ehret et al. However, the patient with the largest tumor volume in our study, i.e., 2.74 cc, had partial paraplegia evaluated at 3/5 and experienced complete recovery of his symptoms. In our cohort, one patient presented with complete paraplegia and was treated with SBRT, to improve neurological functional signs. The general condition and medical contraindications of this patient did not allow for surgical management. The patient died within 9 months of treatment, with no improvement in symptoms. Radiosurgery seems to be most effective in patient who are diagnosed early, with a controlled primary disease, and who present without or minimal neurological symptoms [22, 23].

Studies evaluated in our review have reported heterogeneous treatment patterns. Most studies published in the literature have used a single fraction [13, 15–17] or 3 fraction regimens [9, 11, 17]. In our study, four patients (80%) were treated in six fractions, and one patient was treated in five fractions. Ehret et al. delivered a median total dose of 16 Gy (range: 6–24 Gy) in 1–3 fractions, in mostly single sessions (91%). They reported LC in 64 and 73% at 12 and 24 months, respectively. Four patients (12%) died within 2 months of treatment, from metastatic disease progression [17]. To our knowledge, there are no published data, that have evaluated conventional palliative radiotherapy in the management of IMMs. The treatment regimens found in the literature and performed in our study were different from those performed in palliative radiotherapy (8 Gy in 1 fraction, 20 Gy in 5 fractions, 30 Gy in 10 fractions). In palliative radiotherapy for uncomplicated bone metastasis, the single dose is the regimen of choice [24]. In the case of large soft tissue mass in contact with the bone metastasis, high dose fractionated regimens are recommended [25]. Even if the results are equivalent, the international consensus suggests that single sessions are preferred. SBRT in a single fraction may be an option for patients with IMMs in palliative care, with an altered general condition, in whom it may be necessary to reduce the overall duration of treatment. Nevertheless, previous studies have shown that fractionation increases SC tolerance; however, the optimal balance is not yet known [5]. The higher dose and intra-tumor dose heterogeneity gives rise to the expectation of greater and more durable efficacy. Finally, the cost-effectiveness compared to standard palliative radiotherapy is difficult to assess in this context.

Shin et al. reported that in 9 patients treated (11 IMMs), with a median dose of 14 Gy (range: 10–16 Gy), in 1 fraction, clinical improvement was observed in 80% of patients. They observed complete imaging response in 22% of these patients and partial response in 33%. The median BED_10_ (α/ß = 10) was 33 Gy (20–41.60 Gy) [15]. Nevertheless, the use of the linear-quadratic model for doses per fraction greater than 5–6 Gy is debatable and should be assessed with caution. Veeravagu et al. treated 11 IMMs with CyberKnife®, with a median dose of 21 Gy (range: 14–27 Gy) with a median number of 3 fractions (range: 1–5). They observed clinical improvement in 11% and stable symptomatology in 44% of the patients. They observed no complete response on imaging and partial response in 22%. The BED_10_ delivered was 35.70 Gy, which may explain the results in terms of LC [16]. Barrie et al. delivered a dose of 25 Gy in five fractions to one patient. They observed clinical stability and radiological disease progression (BED_10_ = 37.50 Gy) [14]. Often, radiological follow-up data are not available, and LC is not reported because of the poor prognosis of these pathologies. This makes it difficult to compare the fractionation data and clinical results. Furthermore, our series, which is one of the largest series to date, is the first to describe dosimetric data according to ICRU 91 recommendations.

All lesions in our analysis were treated with CyberKnife®. However, SBRT can be performed using different techniques. According to the ICRU Report 91, SBRT is defined as the administration of high doses per fraction, which must be extremely precise and reproducible [26]. It is an extremely precise irradiation technique, with control of the target and its movements, making it possible to deliver high fractional doses to the tumor by limiting the dose to the organs at risk (OAR) [27, 28]. Hernàndez-Duràn et al., in a systematic review published in 2015, including six studies evaluating SBRT of intramedullary lesions (primary and secondary), reported that the majority of the lesions were treated with CyberKnife® (87%), with no radiation-induced myelopathy in metastatic patients [7]. Radiation-induced myelopathy is a complication of radiation exposure of the SC, which limits the dose that can be administered [5, 6]. The tolerance dose to the spinal cord has been defined based on retrospective series of patients who developed myelopathy from treatment errors or overlapping fields. Baumann et al., evaluated in a review, the incidence of radiation-induced myelopathy after fractioned radiation therapy. At two years of follow up, incidence was 1% in patients treated at 50–55 Gy and 5% when treated at 55–60 Gy [29]. Ryu et al., estimated the partial volume tolerance of the human spinal cord at 10 Gy to the 10% spinal cord volume, defined as 6 mm above and below the target [5]. In our review, we didn’t identify any reports of radiation-induced clinical complications. However, the mean survival should be considered in this context. Indeed, survival is reduced in these patients, which could potentially lead to bias in the identification of radiation-induced complications. Radionecrotic lesions have been described at autopsy in treated patients. These patients did not have any clinical signs or symptoms. This raises the question to know if these anatomical findings have any clinical value [30]. Overall, this review reveals that the delivery of high doses of radiation to a small volume of spinal cord has been safely performed, with a mean prescription dose of 21,6 Gy, yielding good clinical and radiological tumor control.

In our study, two patients (40%) died of disease progression (lung cancer). Ehret et al. reported a median OS of 11.2 months, which is up to five times higher than that reported in other studies [15, 16, 31]. However, in their study, most of the patients were treated for breast cancer and not lung cancer, which may explain the higher OS. Moreover, this finding is also explained by recent improvements in systemic treatments, including immunotherapy and targeted therapies. Despite the diversity of the patients evaluated and the treatments in our study and the heterogeneity of the different studies, it is evident that each case must be evaluated individually, and the treatment modalities were chosen carefully, depending on the patient, disease history, tumor size, localization, and OS. Moreover, the effectiveness of our treatment can be difficult to assess given that histologies were heterogeneous, only two patients had a systemic treatment during SBRT.

The median time from diagnosis of intramedullary metastasis to initiation of radiotherapy was 21 days in our study. Some patients were asymptomatic. However, this treatment is sometimes complex to implement, and the accessibility of this irradiation technique may also be responsible for a delay in management.

Like all the series on this subject, our study had limitations related to its retrospective nature, limited number of patients, and limited follow-up time.

In conclusion, currently, there is no standard of care in SBRT for the management of IMMs. The fractionation scheme is left to the discretion of the physician. The most common patterns found in the literature were 3-fraction (15 Gy in 3 fractions [9], or 27 Gy in 3 fractions [11, 16]) or 5-fraction (25 Gy in 5 fractions [14]) patterns. Ehret et al. proposed most treatments in a single fraction, a short regimen that can be proposed to maintain quality of life. The choice was done depending on the clinical history, the primary lesion (e.g., case of clear cell renal cell carcinoma, typically radioresistant), neurological symptoms, or tumor volume. It seems difficult to define a standard treatment regimen. Although few, if any, neurological complications have been reported, it is essential to find a compromise between the best efficacy and the risk of neurological complications. The palliative setting must be taken into consideration when planning and choosing the treatment. Reducing overall treatment time is an important point to consider not only for patients with multiple comorbidities. Our results showed satisfactory results in terms of LC for patients who often have limited OS. Microsurgical management remains the standard of care, but SBRT is becoming increasingly important in patients with comorbidities as an alternative to surgery. A randomized controlled trial or prospective data are necessary in this context; however, it is difficult to set up in the context of a rare pathology.

## Data Availability

The datasest used and analysed during the current study are available from the corresponding author on reasonable request.
